# ﻿A cryptic species of the *Amolopsricketti* species group (Anura, Ranidae) from China–Vietnam border regions

**DOI:** 10.3897/zookeys.1112.82551

**Published:** 2022-07-14

**Authors:** Jian Wang, Jing Li, Lingyun Du, Mian Hou, Guohua Yu

**Affiliations:** 1 Ministry of Education Key Laboratory for Ecology of Tropical Islands & Key Laboratory of Tropical Animal and Plant Ecology of Hainan Province, College of Life Sciences, Hainan Normal University, Haikou 571158, China; 2 Key Laboratory of Ecology of Rare and Endangered Species and Environmental Protection (Guangxi Normal University), Ministry of Education, Guilin 541004, China; 3 College of Biological and Agricultural Sciences, Honghe University, Mengzi 661199, China; 4 Guangxi Key Laboratory of Rare and Endangered Animal Ecology, College of Life Science, Guangxi Normal University, Guilin 541004, China; 5 College of Continuing (Online) Education, Sichuan Normal University, Chengdu 610068, Sichuan Province, China

**Keywords:** *Amolopsshihaitaoi* sp. nov., *Amolopsyatseni*, new species, Northern Vietnam, Yunnan

## Abstract

It was supposed that the current records of *Amolopsricketti* might be a species complex composed of multiple species. In this study, on the basis of wide sampling, we found that the records of *A.ricketti* from Yunnan, China, and northern Vietnam actually represent a cryptic species based on morphological and molecular evidence. *Amolopsshihaitaoi***sp. nov.** can be distinguished from other members of the *A.ricketti* species group by its moderate body size (SVL 35.5‒37.3 mm in males and 39.2‒45.7 mm in females); white spines on the temporal region, loreal region, snout, and lips in breeding males but absent in females; overlapping heels; tibiotarsal articulation reaching tip of snout; indistinct longitudinal glandular folds on the skin of the shoulders; presence of supernumerary tubercles below the base of fingers II‒IV, distinct pineal body; presence of vomerine teeth; and absence of vocal sacs. Phylogenetic analysis supports that the new species is sister to *Amolopsyatseni* and the populations from Jingxi, Guangxi and Lào Cai, Vietnam previously reported as *A.yatesni* also belong to it. Additionally, our results indicate that more cryptic species may exist within the *A.ricketti* species group, implying that more studies are needed to achieve a complete understanding of the species diversity of this group.

## ﻿Introduction

The cascade frog genus *Amolops* Cope, 1865, which occurs throughout Southeast Asia, southern China, and the southern and eastern Himalayas (Yu et al. 2019; Gan et al. 2020a; Frost 2021), currently contains 73 species (Frost 2021). In China, 42 cascade frog species have been reported (AmphibiaChina 2022), and recently they were assigned into eight species groups, including the *A.monticola* group, *A.chayuensis* group, *A.mantzorum* group, *A.viridimaculatus* group, *A.marmoratus* group, *A.ricketti* group, *A.daiyunensis* group, and *A.hainanensis* group (Jiang et al. 2021).

Generally, members of same species group within *Amolops* share a very similar external adult morphology (e.g. *A.monticola* group), and even some species are more similar in external adult morphology to species of the genus *Odorrana* Fei, Ye & Huang, 1990 (Stuart et al. 2010), which has heavily hampered our understanding of the species diversity in *Amolops* (Wu et al. 2020). During the past two decades, many efforts have been conducted to clarify species diversity within *Amolops* and, notably, a high number of cryptic lineages were discovered. For example, Bain et al. (2003) found six cryptic species in the *Ranachloronota* complex and one of them was later moved to *Amolops* [*A.daorum* (Bain, Lathrop, Murphy, Orlov & Ho, 2003)] by Stuart (2008); Dever et al. (2012) investigated diversity in the *Amolopsmarmoratus* species complex in Myanmar and identified a cryptic species; Lu et al. (2014) suggested that the *A.mantzorum* species group contains five putative species and the nominal species *A.mantzorum* (David, 1872) may in fact include two cryptic species; Fei et al. (2017) recognized the clade consisting of a high-altitude population of the *A.mantzorum* complex in the Yalong river basin as a new species; Jiang et al. (2021) revealed multiple cryptic lineages in the *Amolopschunganensis* complex within *A.monticola* group; and Zeng et al. (2021) found that the populations previously recorded as *A.hongkongensis* (Pope & Romer, 1951) or *A.daiyunensis* (Liu & Hu, 1975) from the coastal hills in eastern Guangdong and southern Fujian represents a cryptic lineage within the *A.daiyunensis* species group. Overall, efforts since 2000 have described more than half of the known species within *Amolops* (Wu et al. 2020), which greatly improves our understanding on the taxonomy and species diversity of this genus.

The *A.ricketti* group is a monophyletic species group containing six recognized species mainly known from southeast China: *A.yunkaiensis* Lyu, Wang, Liu, Zeng & Wang, 2018, *A.albispinus* Sung, Wang & Wang, 2016, *A.wuyiensis* (Liu & Hu, 1975), *A.ricketti* (Boulenger, 1899), *A.sinensis* Lyu, Wang & Wang, 2019, and *A.yatseni* Lyu, Wang & Wang, 2019 (Lyu et al. 2019; Jiang et al. 2021). *Amolopsricketti* was originally described based on specimens from Mount Wuyi, Fujian, China (Boulenger 1899) and had been recorded widely from southern China (i.e. Guangdong, Zhejiang, Jiangxi, Hubei, Hunan, Anhui, and Sichuan; Fei et al. 2012) and northern Vietnam (Nguyen et al. 2009). However, relatively high morphological variation had been observed among populations (Ngo et al. 2006), and recently several cryptic species have been recognized including *A.albispinus*, *A.sinensis*, and *A.yatseni* (Sung et al. 2016; Lyu et al. 2019), indicating that current records of *A.ricketti* might be composed of multiple species and further surveys and studies are required to investigate the species diversity of *A.ricketti* group.

In Yunnan, China, *A.ricketti* has been recorded from Hekou County for over two decades (Yang 1991), but samples of this population have never been included in previous systematic studies. Given that the records of *A.ricketti* from the central region of its geographic range (Hunan, Guizhou, Sichuan, and northeastern Guangxi) have been revised to *A.sinensis* (Lyu et al. 2019; Xiao et al. 2019; Zeng et al. 2021), the records of *A.ricketti* from west region (Yunnan and adjacent Vietnam) probably also need to be revised. Recently, Poyarkov et al. (2021) supposed that *Amolopstonkinensis* (Ahl, 1927 “1926”), a junior synonym of *A.ricketti* described from northern Vietnam, is probably valid, also implying that the taxonomic status of *A.ricketti* from China–Vietnam border regions needs further examination.

During our recent herpetological surveys in Hekou, Yunnan, China, we have collected several *Amolops* specimens previously recorded as *A.ricketti*. Morphological and molecular examinations indicated that these specimens were distinct from *A.ricketti* and other members of the *A.ricketti* group and herein we describe them as a new species.

## ﻿Material and methods

### ﻿Sampling

**Figure 1.**
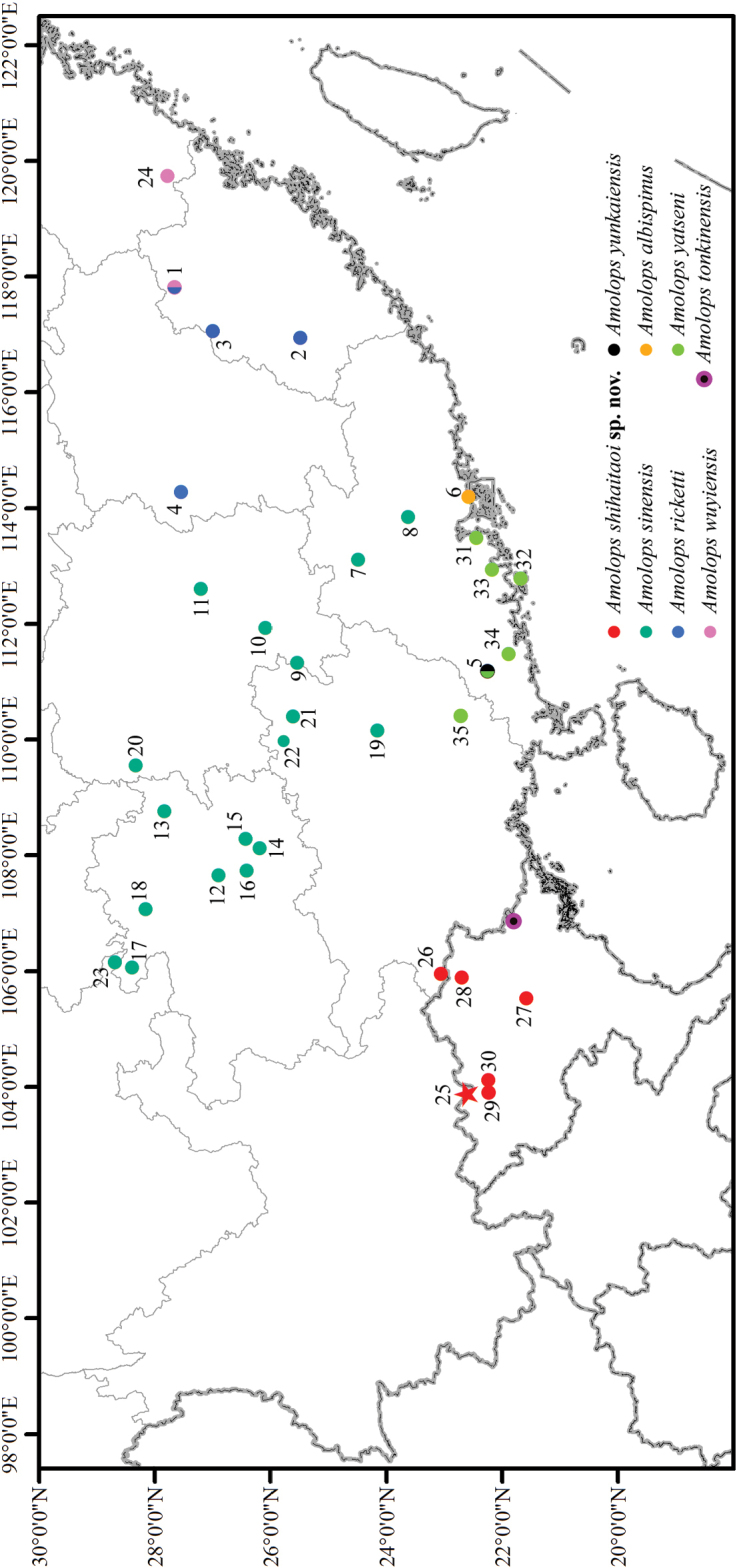
Map showing the collection sites of samples of the *Amolopsricketti* species group used in this study. The red star indicates the type locality of *Amolopsshihaitaoi* sp. nov. and numbers correspond to the locality IDs in Table 1.

Field surveys were conducted in June 2020 at Hekou, Yunnan, China (Fig. 1). Nine specimens were collected, and they were photographed, euthanized, fixed, and then stored in 75% ethanol. Liver tissues were preserved in 99% ethanol. Specimens were deposited at Guangxi Normal University (**GXNU**), Guangxi, China.

### ﻿Morphology

Morphometric data were taken using digital calipers to the nearest 0.1 mm. Morphological terminology follows Yu et al. (2019). Measurements included: snout–vent length (SVL, from tip of snout to vent); head length (HL, from tip of snout to rear of jaws); head width (HW, width of head at its widest point); snout length (SL, from tip of snout to anterior border of eye); internarial distance (IND, distance between nares); interorbital distance (IOD, minimum distance between upper eyelids); upper eyelid width (UEW, maximum width of upper eyelid); eye diameter (ED, diameter of exposed portion of eyeball); nostril-eye distance (DNE, distance from nostril to anterior border of eye); tympanum diameter (TD, the greater of tympanum vertical and horizontal diameters); forearm and hand length (FHL, from elbow to tip of third finger); tibia length (TL, distance from knee to heel); foot length (FL, from proximal end of inner metatarsal tubercle to tip of fourth toe); and length of foot and tarsus (TFL, from tibiotarsal joint to tip of fourth toe). Comparative morphological data of other species in the *A.ricketti* group were taken from their original descriptions or redescriptions (Fei et al. 2009; Sung et al. 2016; Lyu et al. 2018, 2019).

### ﻿Molecular phylogenetic analyses and species delimitation

Total genomic DNA was extracted from liver tissues. Tissue samples were digested using proteinase K, and subsequently purified following a standard phenol/chloroform isolation and ethanol precipitation. Sequences encoding 16S rRNA (16S) and cytochrome oxidase subunit I (COI) genes were amplified using the primers and experimental protocols of Du et al. (2020). PCR amplifications were performed in 50 μl reactions using the following cycling conditions: an initial denaturing step at 95 °C for 4 min; 35 cycles of denaturing at 94 °C for 60 s, annealing at 46 °C (for COI) or 51 °C (for 16S), and extending at 72 °C for 60 s; and a final extension step of 72 °C for 10 min. Sequencing was conducted directly using the corresponding PCR primers. All new sequences were deposited in GenBank (accession no. OK754585‒OK754596 and OK788663–OK788670; Table 1). Available homologous sequences of members of the *A.ricketti* group were obtained from GenBank (Table 1). *Amolopsmengdingensis* Yu, Wu & Yang, 2019, *A.torrentis* (Smith, 1923), *A.hainanensis* (Boulenger, 1900), *A.hongkongensis*, and *A.daiyunensis* were selected as outgroups according to Gan et al. (2020a).

**Table 1.** Samples used in phylogenetic analyses of this study.

Species	Voucher number	Locality (ID)	16s	COI
*A.ricketti*	SYS a4143	Mt. Wuyi, Fujian, China (1)	MK263261	MG991929
	SYS a4142	Mt. Wuyi, Fujian, China (1)	MK263260	MG991928
	SYS a4141	Mt. Wuyi, Fujian, China (1)	MK263259	MG991927
	SYS a4106	Shanghang, Fujian, China (2)	MK263256	MK263311
	SYS a3342	Shanghang, Fujian, China (2)	MK263246	KX507331
	SYS a2492	Mt. Emeifeng, Fujian, China (3)	MK263244	KX507329
	WUSTW01	Mt. Wugong, Jiangxi, China (4)	KF956111	KF956111
*A.yunkaiensis*	SYS a4683	Yunkaishan, Guangdong, China (5)	MK263273	MG991912
	SYS a4684	Yunkaishan, Guangdong, China (5)	MK263274	MG991913
*A.albispinus*	SYS a3452	Mt. Wutong, Guangdong, China (6)	MK263247	KX507332
	SYS a3453	Mt. Wutong, Guangdong, China (6)	MK263248	KX507333
*A.sinensis*	SYS a7106	Shimentai, Guangdong, China (7)	MK263298	MK263330
	SYS a7107	Shimentai, Guangdong, China (7)	MK263299	MK263331
	SYS a5710	Mt. Nankun, Guangdong, China (8)	MK263287	MK263321
	SYS a5089	Dupangling, Guangxi, China (9)	MK263279	MK263319
	SYS a7268	Yangming, Hunan, China (10)	MK263302	MK263334
	SYS a4257	Hengshan, Hunan, China (11)	MK263265	MK263315
	GZNU2018052038	Huangping, Guizhou, China (12)	MN640863	MN643605
	GZNU201805201	Mt. fanjingshan, Guizhou, China (13)	MN640865	MN643607
	GZNU201805001	Danzhai, Guizhou, China (14)	MN640867	MN643609
	GZNU201805002	Leishan, Guizhou, China (15)	MN640868	MN643610
	GZNU201806001	Majiang, Guizhou, China (16)	MN640869	MN643611
	GZNU20170815001	Xishui, Guizhou, China (17)	MN640874	MN643616
	GZNU20170815003	Shuiyang, Guizhou, China (18)	MN640876	MN643618
	YU000067	Mt. Dayao, Guangxi, China (19)	OK754585	‒
	YU000068	Mt. Dayao, Guangxi, China (19)	OK754586	‒
	YU20160156	Jishou, Hunan, China (20)	OK754587	‒
	YU20160406	Xing,an, Guangxi, China (21)	OK754588	‒
	‒	Longshen, Guangxi, China (22)	AY851090	‒
	061001	Hejiang, Sichuan, China (23)	KU840608	‒
	SCUM040518CJ	Hejiang, Sichuan, China (23)	DQ359987	‒
*A.wuyiensis*	SYS a4140	Mt. Wuyi, Fujian, China (1)	MK263258	MK263313
	SYS a4139	Mt. Wuyi, Fujian, China (1)	MK263257	MK263312
	SYS a2723	Jingning, Zhejiang, China (24)	MK263245	MK263303
*A.shihaitaoi* **sp. nov.**	GXNU YU000351	Hekou, Yunnan, China (25)	OK754589	OK788663
	GXNU YU000352	Hekou, Yunnan, China (25)	OK754590	OK788664
	GXNU YU000353	Hekou, Yunnan, China (25)	OK754591	OK788665
	GXNU YU000354	Hekou, Yunnan, China (25)	OK754592	OK788666
	GXNU YU000355	Hekou, Yunnan, China (25)	OK754593	OK788667
	GXNU YU000482	Hekou, Yunnan, China (25)	OK754594	OK788668
	GXNU YU000483	Hekou, Yunnan, China (25)	OK754595	OK788669
	HM 081419	Hekou, Yunnan, China (25)	OK754596	OK788670
	YPX6306	Jingxi, Guangxi, China (26)	MN953758	MN961459
	2000.2938	Tam Dao, Vĩnh Phúc, Vietnam (27)	KR827707	KR087622
	2000.2939	Tam Dao, Vĩnh Phúc, Vietnam (27)	KR827708	KR087623
	ROM26365	Cao Bằng, Cao Bằng, Vietnam (28)	DQ204486	‒
	ROM27276	Sa pa, Lào Cai, Vietnam (29)	MN953723	MN958781
	AMNH168687	Van Ban, Lào Cai, Vietnam (30)	FJ417157	‒
*A.yatseni*	SYS a6806	Zhongshan, Guangdong, China (31)	MK263289	MK263323
	SYS a6807	Zhongshan, Guangdong, China (31)	MK263290	MK263324
	SYS a6808	Zhongshan, Guangdong, China (31)	MK263291	MK263325
	SYS a3633	Shangchuan, Guangdong, China (32)	MK263250	MK263304
	SYS a6818	Gudou, Guangdong, China (33)	MK263294	MK263306
	SYS a3978	Ehuangzhang, Guangdong, China (34)	MK263252	MK263308
	SYS a4642	Yunkaishan, Guangdong, China (5)	MK263269	MK263316
	SYS a7545	Mt. Darong, Guangxi (35)	MZ447966	MZ448269
*A.hongkongensis*	SYS a4577	Hongkong, China	MK263266	MG991919
*A.daiyunensis*	SYS a1739	Mt. Daiyun, Fujian, China	MK263243	KX507328
*A.torrentis*	SYS a5291	Mt. Wuzhi, Hainan, China	MK263286	MG991932
*A.hainanensis*	SYS a5283	Mt. Wuzhi, Hainan, China	MK263283	MG991918
*A.mengdingensis*	KIZ 20160317	Mengding, Yunnan, China	MK501810	MK501813

Sequences were aligned using MUSCLE with the default parameters in MEGA 7 (Kumar et al. 2016). Uncorrected pairwise distances between species were calculated in MEGA 7. Because sequence of COI gene is not available for nine individuals (Table 1), two datasets were prepared for phylogenetic analysis, one including all individuals and one only including individuals for which both two genes are available. The best substitution model of the concatenated data of 16S rRNA and COI genes was selected using the Akaike Information Criterion (AIC) in MODELTEST v. 3.7 (Posada and Crandall 1998). Bayesian inferences were performed in MRBAYES v. 3.2.6 (Ronquist et al. 2012) under the selected substitution model (GTR + I + G). Two runs were performed simultaneously with four Markov chains starting from random tree. The chains were run for 3,000,000 generations and sampled every 100 generations. The first 25% of the sampled trees were discarded as burn-in after the standard deviation of split frequencies of the two runs was less than a value of 0.01, and then the remaining trees were used to create a consensus tree and to estimate Bayesian posterior probabilities (BPPs).

We used the method of Assemble Species by Automatic Partitioning (ASAP; Puillandre et al. 2021) to attempt to delimit the species boundary among the *A.ricketti* species group based on the combined data of 16S rRNA and COI sequences. For this analysis, the substitution model of simple distance (*p*-distances) was selected and the partitioning with lowest ASAP-score was selected as the best according to Puillandre et al. (2021).

## ﻿Results

**Figure 2.**
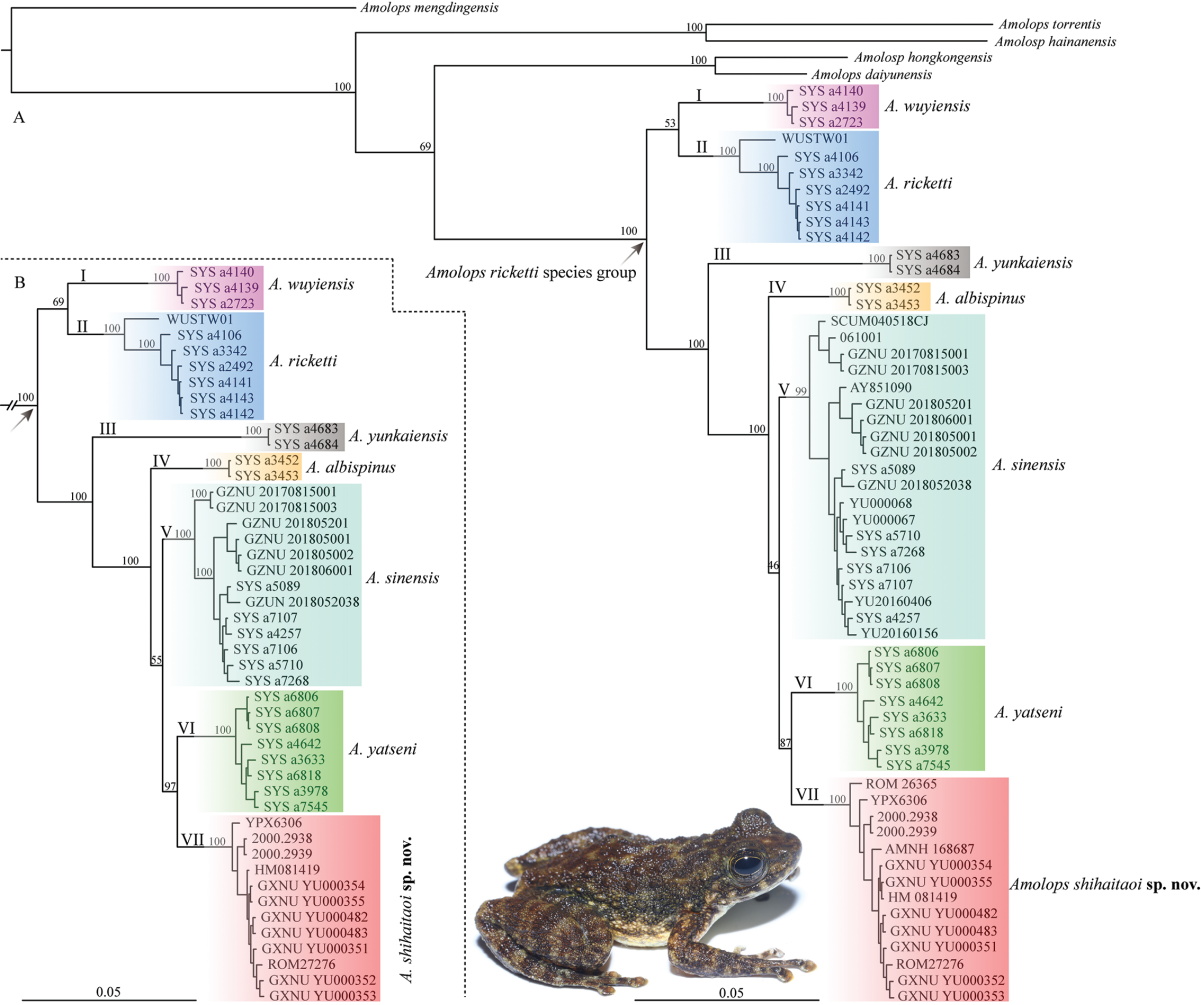
Bayesian phylogram of the *Amolopsricketti* species group inferred from the combined data of 16S and COI genes with inclusion of all individuals (**A**) and inclusion of only individuals for which both the two genes are available (**B**).

The obtained 16S and COI alignments were 1036 and 667 bp, respectively. The *A.ricketti* group was a monophyletic species group containing seven well-supported distinct clades, of which six (Clades I‒VI) correspond to the six known species of this group. The clade VII is comprised of populations previously recorded as *A.ricketti* from Yunnan and Vietnam and a specimen previously classified as *A.yatseni* from Jingxi, Guangxi, China (YPX6306), and it was the sister to *A.yatseni* (Fig. 2). The genetic divergences between clade VII and *A.yatseni* estimated from 16S and COI genes are 1.9% and 5.7%, respectively (Table 2).

**Table 2.** Uncorrected pairwise distances among members of the *A.ricketti* species group estimated from 16S rRNA (lower triangle) and COI sequences (upper triangle).

	Species	1	2	3	4	5	6	7
1	*A.shihaitaoi* **sp. nov.**		0.057	0.061	0.064	0.102	0.101	0.104
2	*A.yatseni*	0.019		0.066	0.072	0.105	0.100	0.103
3	*A.sinensis*	0.020	0.019		0.060	0.099	0.101	0.111
4	*A.albispinus*	0.021	0.025	0.025		0.096	0.108	0.104
5	*A.yunkaiensis*	0.048	0.049	0.046	0.052		0.118	0.113
6	*A.wuyiensis*	0.036	0.041	0.035	0.046	0.058		0.095
7	*A.ricketti*	0.040	0.046	0.043	0.044	0.058	0.028	

**Figure 3.**
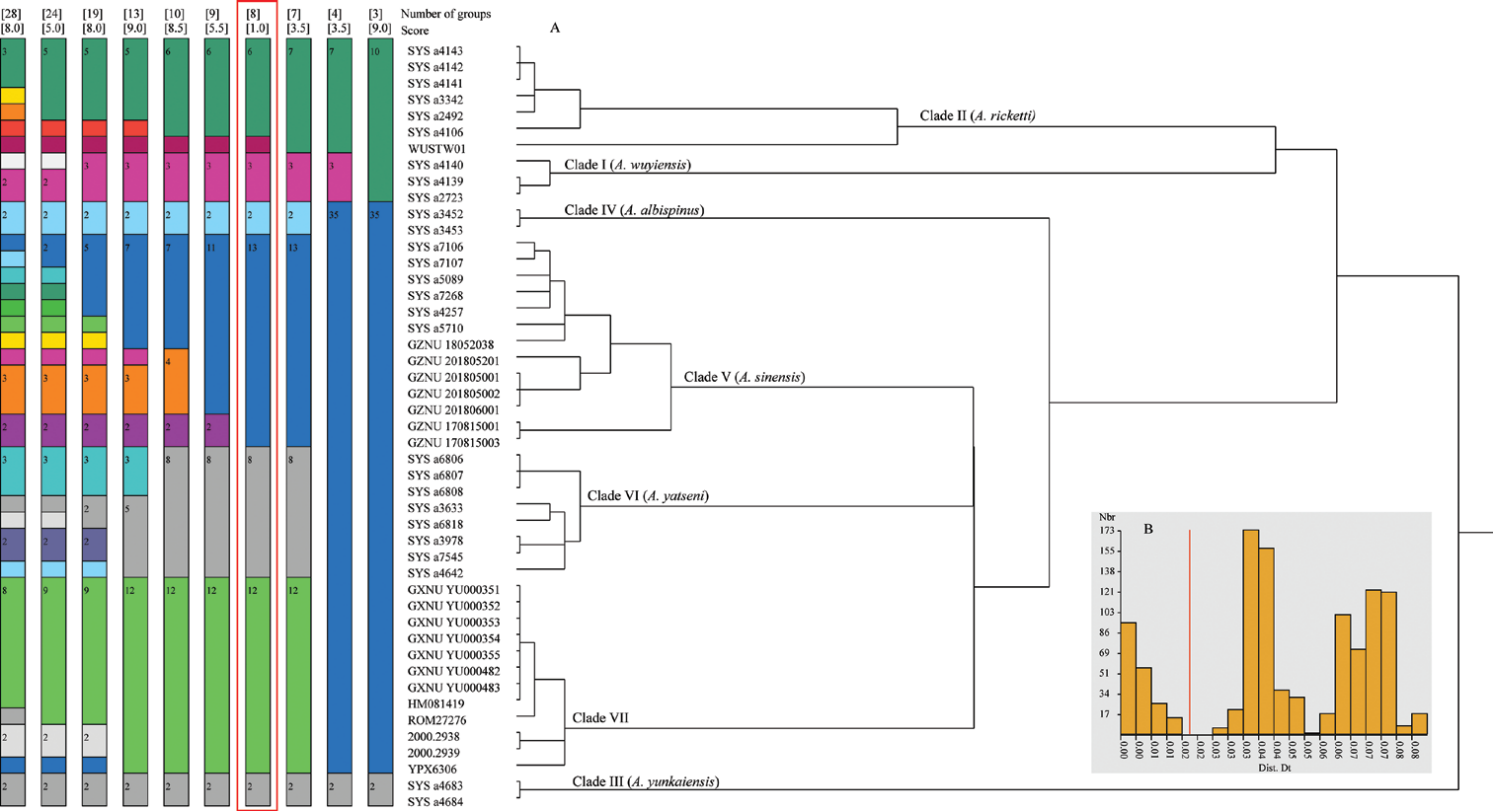
ASAP species delimitation within the *A.ricketti* species group based on the combined data of 16S and COI sequences. The best partition with lowest score is highlighted with red frame.

The analysis of species delimitation based on the combined data found 10 partitions (Fig. 3a). The best partition (score = 1.00) grouped the samples into eight species with a distance threshold of c. 2% (Fig. 3b) and one of them corresponds to the clade consisting of the samples from Yunnan and northern Vietnam (Clade VII). All other clades were recognized as distinct species by the ASAP analysis with the exception of clade II, which was grouped into two different species, one containing the samples of *A.ricketti* from Fujian (including the type locality) and one containing the sample of *A.ricketti* from Mount Wugong, Jiangxi, China (WUSTW01).

Morphologically the specimens from Hekou, Yunnan, China were distinguished from all other recognized members of the *A.ricketti* group by a series of characters. Thus, we consider that the clade VII represents a distinct species. Ahl (1927 “1926”) once described *Rhacophorustonkinensis* Ahl, 1927 “1926” based on one specimen from Tonkin (probably Mau Son, Lang Son Province, Vietnam according to Bourret [1942]; Fig. 1), but later Bourret (1942) regarded it to be a junior synonym of *A.ricketti*. Recently, Poyarkov et al. (2021) supposed that *A.tonkinensis* should be treated as a distinct species or as a senior synonym of *A.yatseni*. Body size of the type of *A.tonkinensis* (sex unknown) is 56 mm (Ahl 1927 “1926”), which is obviously larger than our specimens from Hekou, Yunnan, China in body size (35.5‒37.3 mm in males and 39.2‒45.7 mm in females). In addition, specimens from Hekou differ from *A.tonkinensis* by tibiotarsal articulation reaching tip of snout and upper eyelid width greater than interorbital space (vs tibiotarsal articulation reaching central of eye and upper eyelid width equal to interorbital space; Ahl 1927 “1926”). Therefore, we consider that the clade VII is not conspecific with the nomen *A.tonkinensis* and describe it as new.

### ﻿ Amolops shihaitaoi sp. nov.

Taxon classificationAnimaliaAnuraRanidae

5E7F2729-FFCD-518D-B86E-6B1571486D20

https://zoobank.org/025A83B3-B0EE-4632-9284-3BA7175CAA6E

Figs 4 , 5 , 6 , 7

#### Chresonymy.

*Amolopsricketti* in Yang (1991), Inger et al. (1999), Ngo et al. (2006), Yang and Rao (2008), Nguyen et al. (2009), Stuart et al. (2010), Grosjean et al. (2015); *Amolopsyatseni* in Wu et al. (2020); *Amolopstonkinensis* in Poyarkov et al. (2021).

#### Holotype.

**Figure 4.**
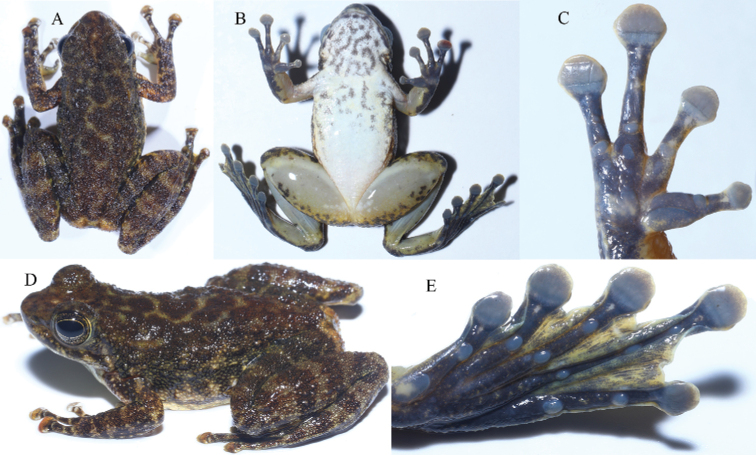
Views of the holotype of *Amolopsshihaitaoi* sp. nov. (GXNU YU000353) in life.

**Figure 5.**
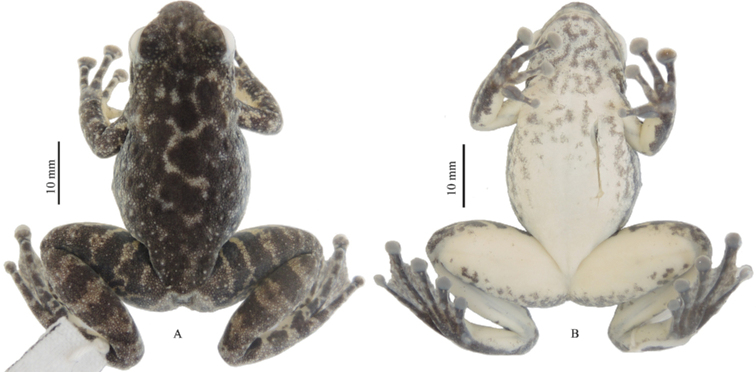
Holotype of *Amolopsshihaitaoi* sp. nov. (GXNU YU000353) in preservative **A** dorsal view **B** ventral view.

GXNU YU000353 (Figs 4, 5), adult female, collected on 21 June 2020 by Jian Wang from Hekou, Yunnan, China (22.6287°N, 103.8776°E; 532 m a.s.l.).

#### Paratypes.

Six adult females (GXNU YU000351, GXNU YU000352, GXNU YU000354, GXNU YU000355, GXNU YU000478, and GXNU YU000479) and two adult males (GXNU YU000482 and GXNU YU000483) with same collection information as holotype.

#### Etymology.

Specific epithet *shihaitaoi* is named after Prof. Hai-Tao Shi from Hainan Normal University for his outstanding contribution to the herpetology of China. We suggest the common English name “Hekou torrent frog” and Chinese name “Hé Kǒu Tuān Wā (河口湍蛙)”.

#### Diagnosis.

The new species is assigned to genus *Amolops* and further to the *A.ricketti* group morphologically based on the absence of dorsolateral folds, presence of circummarginal groove on disc of the first finger, disc of first finger distinctly smaller than that of second finger, absence of tarsal fold and tarsal glands, and presence of nuptial pads with conical nuptial spines on the first finger in breeding male.

*Amolopsshihaitaoi* sp. nov. can be distinguished from other members of *A.ricketti* group by having a combination of the following characters: body size moderate (SVL 35.5‒37.3 mm in males and 39.2‒45.7 mm in females); white spines on temporal region, loreal region, snout, and lips present in breeding males but absent in females (Fig. 5); presence of small, dense, translucent or white spines on the dorsal skin of the body, dorsal and dorsolateral skin of limbs; heels overlapping; tibiotarsal articulation reaching tip of snout; longitudinal glandular folds on the skin of shoulders indistinct; presence of supernumerary tubercles below the base of fingers II‒IV, pineal body distinct; presence of vomerine teeth; and absence of vocal sacs.

#### Description of holotype.

**Adult female** (SVL 43.8 mm; Table 3); head width (HW 15.2 mm) greater than head length (HL 13.6 mm; HW/HL = 1.12); snout short and rounded in profile, projecting beyond margin of lower jaw in ventral view; canthus rostralis distinct; loreal region sloping, concave; nostrils oval, lateral; internarial distance (IND 5.5 mm) greater than interorbital distance (IOD 3.3 mm; IND/IOD = 1.67); upper eyelid width (UEW 4.3 mm) greater than interorbital space (UEW/IOD = 1.30); pineal spot present; pupil oval, horizontal; tympanum small (TD 1.5 mm), rounded, less than half eye diameter (ED 5.9 mm; TD/ED = 0.25); supratympanic fold distinct, start from posterior edge of eye and extending to should; vomerine teeth in two oblique rows between choanae; choanae oval; tongue cordiform, deeply notched posteriorly.

**Table 3.** Measurements (in mm) of *Amolopsshihaitaoi* sp. nov. from the type locality (Holotype is marked with asterisk; M: male; F: female).

Voucher no.	sex	SVL	HL	HW	SL	IND	IOD	UEW	ED	TD	DNE	FHL	TL	TFL	FL
GXNU YU000351	F	39.2	13.3	14.9	5.2	5.5	3.3	3.9	5.2	1.6	2.8	20.4	22.7	30.0	20.3
GXNU YU000352	F	43.2	13.4	15.3	5.2	5.5	3.6	4.1	5.5	1.6	3.0	21.6	24.5	33.4	21.8
GXNU YU000353^*^	F	43.8	13.6	15.2	5.3	5.5	3.3	4.3	5.9	1.5	2.8	20.5	22.7	30.7	20.9
GXNU YU000354	F	39.4	12.7	14.8	5.1	5.2	3.3	4.3	5.5	1.5	2.7	20.8	23.2	30.9	20.2
GXNU YU000355	F	45.7	14.3	15.5	5.9	5.7	4	4.1	5.3	1.5	2.4	21.6	23.7	33.5	22.4
GXNU YU000478	F	45.1	13.9	16.2	5.9	5.6	3.9	4.2	5.7	1.8	2.5	22.5	25.2	32.5	22.6
GXNU YU000479	F	45.3	13.6	15.7	5.5	5.4	3.2	4.2	5.7	1.9	2.7	22.0	25.2	33.5	22.2
GXNU YU000482	M	37.3	11.7	13.4	4.8	5.3	2.7	3.7	5.0	1.8	2.2	19.3	21.8	29.6	19.7
GXNU YU000483	M	35.5	10.9	13.2	4.7	5.0	3.0	3.1	4.9	1.6	1.9	19.2	21.1	28.5	18.4

Forelimbs moderately robust; relative length of fingers I<II<IV<III; all fingertips expanded into discs with circummarginal grooves, relative width of finger disks I<II<III=IV; webbing between fingers absent; subarticular tubercles prominent and rounded, formula 1, 1, 2, 2; supernumerary tubercle present and prominent below the base of fingers II‒IV; two metacarpal tubercles.

Hindlimbs long and robust, tibiotarsal articulation reaching tip of snout when hindlimb stretched alongside of body; heels slightly overlapping when legs positioned at right angles to body; tarsal glands absent; relative length of toes I<II<III=V<IV; all toe tips expanded into discs with circummarginal grooves; toes fully webbed, webbing formula I1-1II1-1III1-1IV1-1V; lateral fringes of toes I and V developed; subarticular tubercles prominent and rounded, formula 1, 1, 2, 3, 2; inner metatarsal tubercle prominent; outer metatarsal tubercle absent.

Dorsolateral fold absent; dorsal surface rough and granular with denser small translucent or white warts on dorsal body and dorsal limbs; flanks very rough and granular, scattered with large raised white tubercles; rictal gland prominent; large white and small translucent warts present around the vent; skin of throat, chest, and venter slightly wrinkled, both sides of venter obviously granular; ventral surface of limbs smooth.

#### Coloration in life.

Dorsal surface of olive-brown with dark brown patches on dorsal surface of head and trunk and dark brown irregular transverse bars on dorsal surface of limbs; dorsal surface of discs white-mottled with cropper on discs of fingers III and IV and all toe discs; region around cloaca olive-brown with rusty mottling on both sides; sides of head olive-brown with dark brown blotches; rictal gland light yellow; flanks olive-brown, warts on flanks dark or white; throat and chest creamy white scattered with distinct dark blotches and mottled with light yellow; belly creamy white mottled with light yellow; ventral surface of limbs semi-opaque, grey, mottled with light yellow; webbing between toes beige, mottled with black; iris black with brown mottling (Fig. 4).

#### Coloration in preservative.

Dorsal surface dark brown, with irregular light patches; dark brown transverse bars on limbs distinct; ventral surface grayish white, with dark mottling on throat and chest (Fig. 5).

#### Morphological variation.

**Figure 6.**
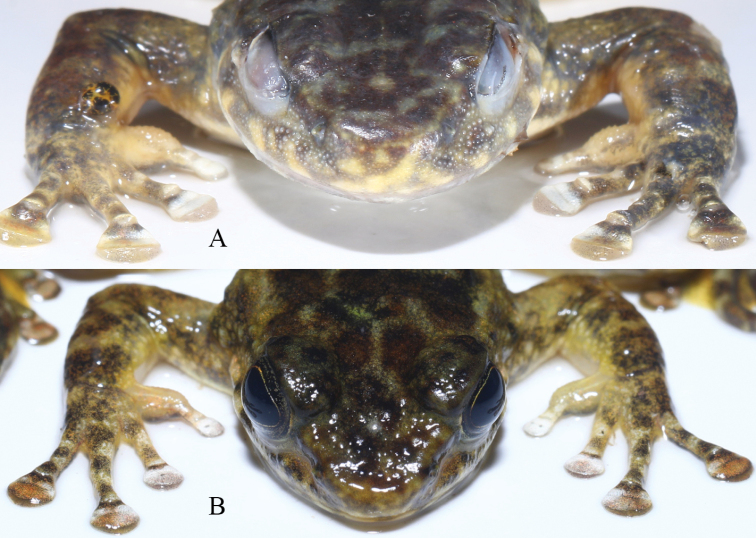
Two male paratypes of *Amolopsshihaitaoi* sp. nov. **A** GNXU YU000483 **B**GXNU YU000482.

**Figure 7.**
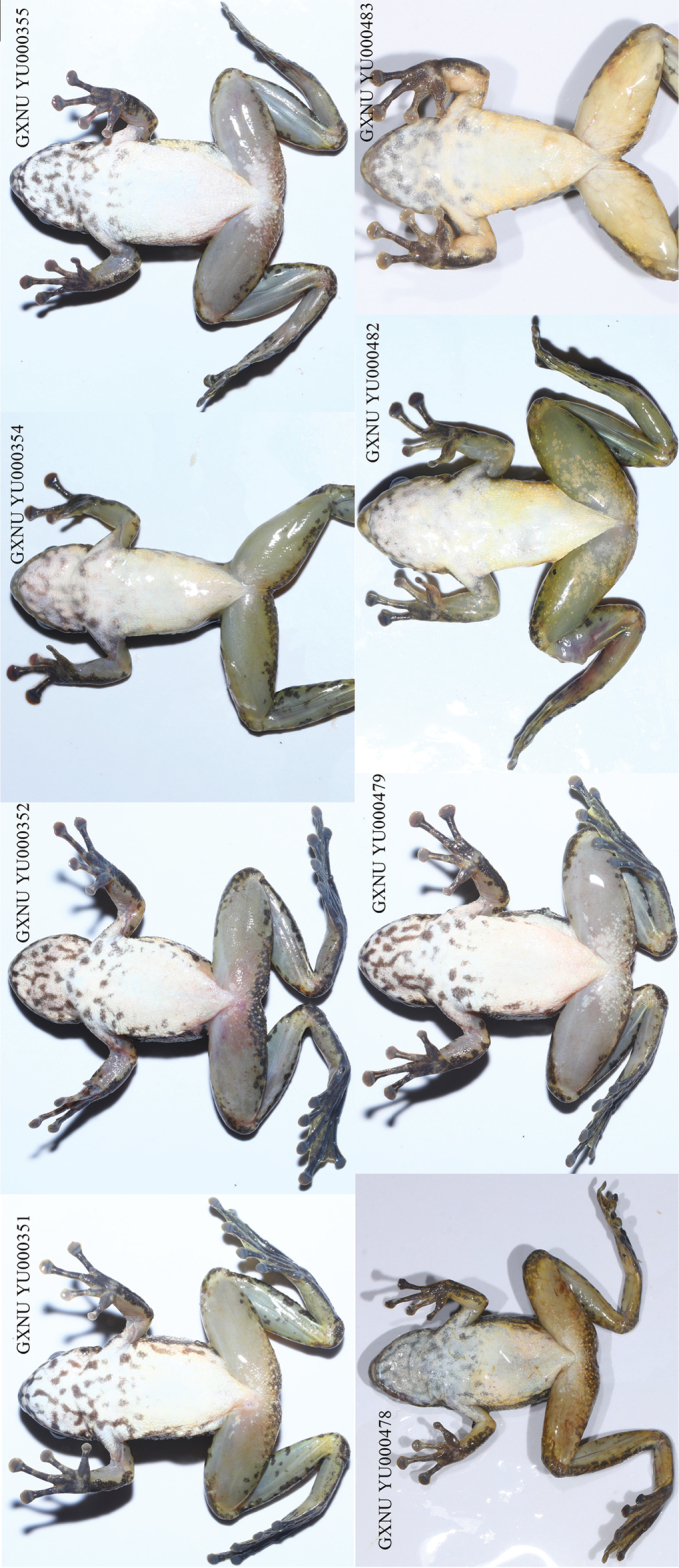
Ventral view of paratypes of *Amolopsshihaitaoi* sp. nov. in life.

The new species is sexually dimorphic. Males are smaller than females (Table 1) and possess nuptial pads with spines in the breeding season (Fig. 6). Additionally, spines on dorsal skin in males are less distinct than in females. A male specimen in breeding season (GXNU YU000483) has distinct spines on the temporal region, loreal region, snout, lips, and chin, and has conical spines on the nuptial pad, whereas a male specimen in the early stage of development (GXNU YU000482) lacks distinct spines on the temporal region, loreal region, snout, lips, and chin and its nuptial spines are papillate (Fig. 6). All types have no beige snowflake-like patches on the ventral surface of limbs with the exception of GXNU YU000482. In addition, the three types (GXNU YU000352, GXNU YU000353, GXNU YU000355, and GXNU YU000479) nearly have no light yellow coloration of on the undersides of the limbs. Compared to other types, GXNU YU000482 has less distinct dark patches on the throat and chest (Fig. 7).

#### Distribution.

**Figure 8.**
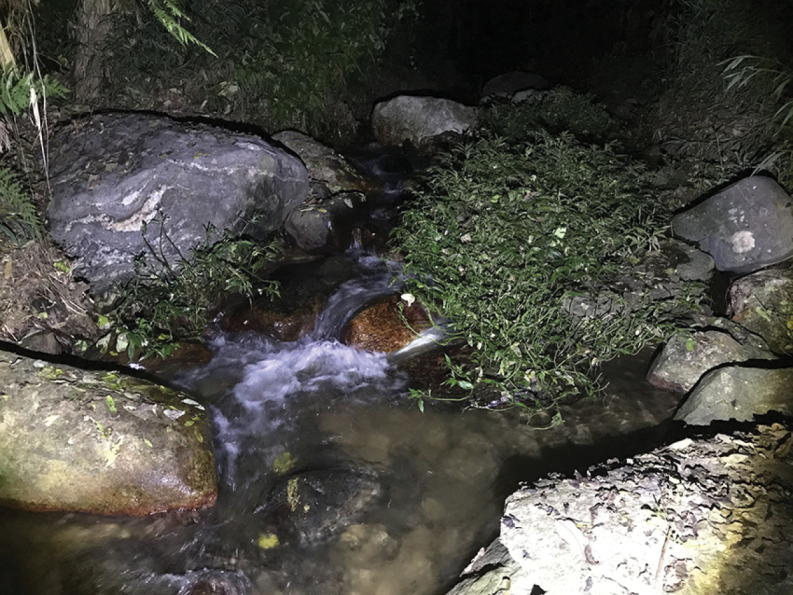
Habitat of *Amolopsshihaitaoi* sp. nov. at the type locality.

In addition to the type locality in Hekou, Yunnan, China, the new species also occurs in Jingxi, Guangxi, China and northern Vietnam (Vĩnh Phúc, Cao Bằng, and Lào Cai) because our molecular analyses revealed that samples from Jingxi, Vĩnh Phúc, Cao Bằng, and Lào Cai that were sequenced by previous studies also belong to the new species. In Yunnan, the new species inhabits rocky streams (Fig. 8). Much of the ecology and behavior of this species remains unknown.

#### Comparisons.

**Figure 9.**
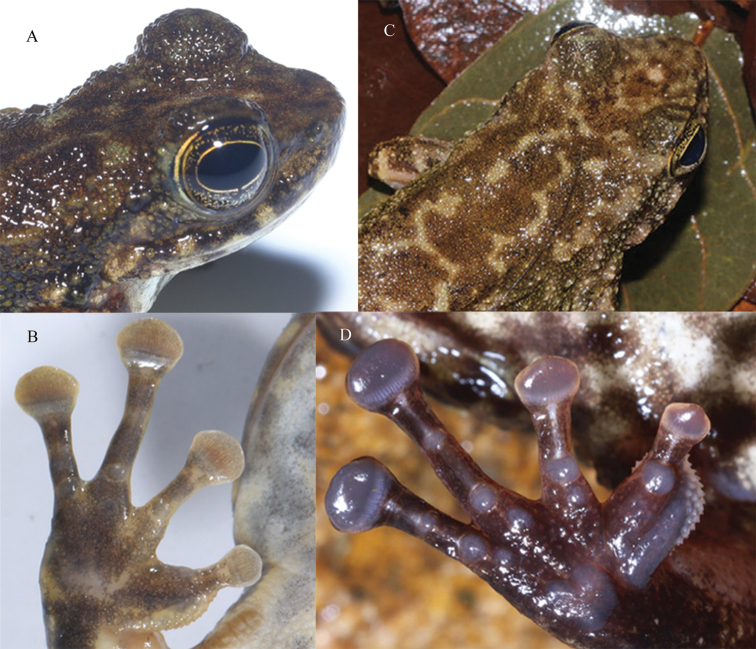
**A, B***Amolopsshihaitaoi* sp. nov. **A** head of female holotype GXNU YU000353 **B** hand of male paratype GXNU YU000483 **C, D***A.yatseni* (reproduced from Lyu et al. 2019) **C** head of female paratype SYS a003981 **D** hand of male holotype SYS a006807.

The absence of dorsolateral folds, presence of circummarginal groove on disc of the first finger, disc of first finger distinctly smaller than that of second finger, absence of tarsal fold and tarsal glands, and presence of nuptial pads with conical nuptial spines on the first finger in breeding males suggest that the new species belongs to the *A.ricketti* species group, which is supported by the molecular evidence (Fig. 2). Morphological comparisons among the members of the *A.ricketti* species group are summarized in Table 4. *Amolopsshihaitaoi* sp. nov. is recovered as the sister taxon to *A.yatseni*, but morphologically it differs from the later by the absence of white spines on temporal region and lower lips in female (vs present; Fig. 9), supernumerary tubercles moderate developed (vs very distinct; Fig. 9), and heels overlapping (vs just meeting).

**Table 4.** Morphological comparison between members of the *A.ricketti* species group. Characters are: ① vomerine teeth: 0 = absent, 1 = present; ② vocal sacs: 0 = absent, 1 = present; ③ spines on temporal region and lower lips in female: 0 = absent, 1 = present; ④ heels: 0 = overlapping, 1 = just meeting; ⑤ translucent or white spines on dorsal body, dorsal and dorsolateral limbs: 0 = absent, 1 = present; ⑥ spines on temporal region, loreal region, and lips in breeding male: 0 = absent, 1 = present; ⑦ tibiotarsal articulation: 0 = reaching tip of snout, 1 = reaching eye; ⑧ supernumerary tubercle below the base of fingers II: 0 = absent, 1 = present; ⑨ pineal body: 0 = distinct, 1 = indistinct; ⑩ female SVL (mm). “?” = unknown.

Species	①	②	③	④	⑤	⑥	⑦	⑧	⑨	⑩	Source
*A.shihaitaoi* **sp. nov.**	1	0	0	0	1	1	0	1	0	39.2‒45.7	This study
*A.yatseni*	1	0	1	1	1	1	0	1	0	42.1–48.9	a
*A.ricketti*	1	0	0	0	0	0	1	0	0	53.5‒67.0	a, b
*A.sinensis*	1	0	0	0	0	1	0	0	0	47.7–52.7	a
*A.albispinus*	1	0	0	?	0	1	0	0	1	43.1–50.9	a, b
*A.wuyiensis*	0	1	0	0	0	0	0	1	0	45.2–52.7	a, b, c
*A.yunkaiensis*	0	1	0	0	0	0	0	1	1	35.2–39.0	a, d

Source: a: Lyu et al. (2019); b: Sung et al. (2016); c: Fei et al. (2009); d: Lyu et al. (2018).

The new species has been previously reported as *A.ricketti*, but it can be distinguished from the later by smaller body size (39.2‒45.7 mm vs 53.5‒67.0 mm in females), presence of dense small translucent or white spines on the dorsal skin of the body, dorsal and dorsolateral skin of limbs (vs absent), presence of spines on skin of temporal region, loreal region, and lips in breeding males (vs absent), and tibiotarsal articulation reaching tip of snout (vs reaching eyes). *Amolopsshihaitaoi* sp. nov. differs from *A.sinensis* by relatively smaller body size (SVL 39.2‒45.7 mm vs 47.7‒52.7 mm in females), presence of small, dense, translucent or white spines on the dorsal skin of the body, dorsal and dorsolateral skin of limbs (vs absent), presence of supernumerary tubercles below the base of finger II (vs absent), and longitudinal glandular folds on the skin of shoulders indistinct (vs distinct); from *A.albispinus* by the presence of dense translucent or white spines on the dorsal skin of the body, dorsal and dorsolateral skin of the limbs (vs absent), ventral surface relatively smooth (vs with numerous small tubercles and ridges on the throat and ventral surfaces of trunk and limbs), and pineal body distinct (vs indistinct); and from *A.wuyiensis* and *A.yunkaiensis* by the presence of vomerine teeth (vs absent) and absence of vocal sacs (vs present). The new species further obviously differs *A.wuyiensis* by nuptial spines beige (vs black).

## ﻿Discussion

*Amolopsricketti* was once recorded widely from southern China and Indochina (Fei et al. 2012), but recent studies based on samples mainly from east and middle parts of its distribution range showed that it actually contains multiple cryptic species (Sung et al. 2016; Lyu et al. 2019; Xiao et al. 2019), indicating that a taxonomic investigation for the records from west part of its distribution range is needed to precisely determine the species diversity and distribution of the *A.ricketti* species group. In this study, we found that the populations from Yunnan and Vietnam previously recorded as *A.ricketti* represent a distinct species based on morphological and molecular evidence, and the population from Jingxi, Guangxi, which was recently classified as *A.yatseni* in Wu et al. (2020), also belongs to it. This finding further improves our understanding of the taxonomy and distribution of the *A.ricketti* species group (Fig. 1).

The taxonomy of *A.ricketti* species group in northern Vietnam is complicated and needs further study. Recently, *A.tonkinensis*, a junior synonym of *A.ricketti* described from Tonkin (probably Lang Son Province of Vietnam according to Bourret [1942]), was considered probably valid or a senior synonym of *A.yatseni* by Poyarkov et al. (2021). We think that *A.shihaitaoi* sp. nov. is not conspecific with *A.tonkinensis* because the new species described here has smaller body size, longer hindlimbs, and upper eyelid width greater than interorbital space as we mentioned above. Additionally, Pham et al. (2020) reported that males of *A.ricketti* from Hai Ha forest, Quang Ninh Province, Vietnam have vocal sacs, which is questionable and needs further confirmation because *A.ricketti* sensu stricto, *A.sinensis*, *A.yatseni*, and *A.shihaitaoi* sp. nov. have no vocal sacs. It is possible that the record of *A.ricketti* from Quang Ninh represents a cryptic species or refers to *A.tonkinensis*. In addition, the taxonomic status of *A.ricketti* from Mount Wugong, Jiangxi also needs further investigation because the sample from this locality was not grouped into same species with *A.ricketti* from the type locality by the analysis of species delimitation. In summary, more cryptic species may exist within the *A.ricketti* species group, implying that more studies are needed to achieve a complete understanding on the species diversity of this group.

The genus *Amolops* is the most diverse group of ranid frogs and currently contains 74 species including *A.shihaitaoi* sp. nov. Of these 74 species, more than a third (30) were described in the last decade (2010‒2021), indicating that the species diversity of *Amolops* was highly underestimated and the taxonomy of *Amolops* has attracted much attention of herpetologists during the last decade (e.g. Stuart et al. 2010; Dever et al. 2012; Sun et al. 2013; Jiang et al. 2016; Fei et al. 2017; Yuan et al. 2018; Yu et al. 2019; Che et al. 2020; Gan et al. 2020a, 2020b; Zeng et al. 2021). Most of the newly named *Amolops* species during the last ten years are from China (especially southwestern China), reflecting high species diversity of *Amolops* in China and probably the shortage of amphibian surveys in adjacent countries (e.g. Myanmar; Gan et al. 2020b).

Yunnan is the region with highest amphibian species diversity in China (AmphibiaChina 2022), and in recent years a number of new species or newly recorded *Amolops* were discovered from Yunnan (e.g. Yuan et al. 2018; Yu et al. 2019; Gan et al. 2020a; Wu et al. 2020). Including the new species described here, up to now there are 14 *Amolops* species known from Yunnan, including *A.afghanus* (Günther, 1858), *A.bellulus* Liu, Yang, Ferraris & Matsui, 2000, *A.daorum*, *A.jinjianensis* Su, Yang & Li, 1986, *A.loloensis* (Liu, 1950), *A.mantzorum*, *A.mengdingensis*, *A.mengyangensis* Wu & Tian, 1995, *A.shihaitaoi* sp. nov., *A.splendissimus* Orlov & Ho, 2007, *A.tuanjieensis* Gan, Yu & Wu, 2020, *A.tuberodepressus* Liu & Yang, 2000, *A.viridimaculatus* (Jiang, 1983), and *A.wenshanensis* Yuan, Jin, Li, Stuart & Wu, 2018. *Amolopschunganensis* (Pope, 1929) was once recorded from Jinghong and Menglian in Yunnan (Yang and Rao 2008), but these two records should be revised to *A.mengyangensis* and *A.tuanjieensis*, respectively (Jiang et al. 2021).

Compared to Yunnan, the species diversity of *Amolops* in Guangxi is much lower and currently only five species are exactly known from there including *A.chunganensis* (Jiang et al. 2021), *A.sinensis* (Lyu et al. 2019; this study), *A.wenshanensis* (Yuan et al. 2018), *A.yatseni* (Zeng et al. 2021), and the new species described here.

### ﻿A key to the members of *Amolopsricketti* species group

**Table:** 

1	Vomerine teeth absent and vocal sac present	**2**
–	Vomerine teeth present and vocal sac absent	**3**
2	Nuptial spines black	***A.wuyiensis***
–	Nuptial spines white	***A.yunkaiensiis***
3	Breeding male lacks spines on temporal region, loreal region, and lips	***A.ricketti***
–	Breeding male has spines on temporal region, loreal region, and lips	**4**
4	Pineal body barely visible	***A.albispinus***
–	Pineal body very distinct	**5**
5	Translucent or white spines absent on dorsal body, dorsal and dorsolateral limbs and supernumerary tubercle below the base of fingers II absent	***A.sinensis***
–	Translucent or white spines present on dorsal body, dorsal and dorsolateral limbs and supernumerary tubercle below the base of fingers II present	**6**
6	Spines present on temporal region and lower lips in female and heels just meeting	***A.yatseni***
–	Spines absent on temporal region and lower lips in female and heels overlapping	***A.shihaitaoi* sp. nov.**

## Supplementary Material

XML Treatment for Amolops shihaitaoi
